# Modulation Effects of Reproductive Hormones on Oogenesis in a Collagenase-Induced Osteoarthritis Mouse Model

**DOI:** 10.3390/biomedicines14040857

**Published:** 2026-04-09

**Authors:** Anton Kolarov, Irina Chakarova, Valentina Hadzhinesheva, Venera Nikolova, Stefka Delimitreva, Maya Markova, Ralitsa Zhivkova

**Affiliations:** Department of Biology, Medical Faculty, Medical University of Sofia, 2 Zdrave Str, 1431 Sofia, Bulgaria; a.kolarov@medfac.mu-sofia.bg (A.K.); i.chakarova@medfac.mu-sofia.bg (I.C.); v.hadzhinesheva@medfac.mu-sofia.bg (V.H.); v.nikolova@medfac.mu-sofia.bg (V.N.); s.delimitreva@medfac.mu-sofia.bg (S.D.); m.markova@medfac.mu-sofia.bg (M.M.)

**Keywords:** collagenase-induced osteoarthritis, oogenesis, meiotic spindle, actin cap, estradiol, follicle-stimulating hormone

## Abstract

**Background/Objectives**: Osteoarthritis has been increasingly described as associated with systemic inflammation, raising the question of how it would affect fertility in young women with or without reproductive hormone administration. We studied oogenesis in mice with collagenase-induced osteoarthritis (CIOA) as a model system with fewer ethical limitations after estradiol (E2) or follicle-stimulating hormone (FSH) treatment. **Methods**: Oocytes have been isolated from mice subjected to various treatment regimens. The meiotic spindle, the chromatin, and the actin cap were fluorescently labeled and analyzed. **Results**: In addition to reduced maturation rates, specific oocyte abnormalities were registered when CIOA, FSH, or E2 were applied in isolation. Combined treatments showed that the spindle, chromatin, and actin cytoskeleton parameters were differently affected in oocytes from groups with CIOA treated by estradiol and those treated with FSH. Enlarged spindles, ooplasmic tubulin asters, aligned metaphases, and predominantly normal actin caps, often with an actin halo, were typical for groups with CIOA combined with estradiol. The groups with CIOA and FSH had slightly enlarged spindles, unaligned metaphases with degenerated chromatin surrounded by a cloud of depolymerized tubulin, and small actin caps. **Conclusions**: Our results show that experimental osteoarthritis with or without exogenous reproductive hormones negatively affects oogenesis, presumably due to systemic inflammatory factors making the ovarian microenvironment less capable of supporting oocyte maturation. Estradiol supplementation does not benefit oogenesis. FSH treatment induced cytoskeletal and chromatin abnormalities that presumably disturb the fertilization and development potential of affected oocytes. These data can have implications for assisted reproduction in cases of patients with osteoarthritis.

## 1. Introduction

Oogenesis is a process with a complex and ambiguous relationship to inflammation. On the one hand, inflammation-like changes in the ovary have been shown to be a prerequisite for ovulation [1,2]. The surge of pituitary-derived luteinizing hormone stimulates the somatic cells of the Graafian follicle to produce prostaglandins and cytokines. In response to these mediators of inflammation, neighboring cells release proteases that digest the extracellular matrix. This weakens the wall of the Graafian follicle and the surrounding tissue and allows its rupture [1,2]. Anti-inflammatory drugs, if applied in a critical time window, interfere with ovulation in both women [3] and animal models [4].

On the other hand, inflammation can have adverse effects on female reproductive functions. Pathological conditions such as obesity, polycystic ovary syndrome, and endometriosis, which disturb oogenesis and ovulation and diminish female fertility, are increasingly described as associated with chronic low-grade inflammation, which is essential for their negative impact on reproduction [1,5,6]. The treatment of resulting infertility is a difficult challenge. Because it is usually not possible to eliminate the root causes, efforts are concentrated on dealing with the consequences. Anti-inflammatory drugs can help, particularly in endometriosis [7], but they are not expected to achieve success on their own; moreover, they should be used with caution, given the above-cited data about the mechanism of ovulation. The complex crosstalk between endocrine factors and the underlying pathological processes means that there are no straightforward, universal recommendations; rather, the treatments establishing a suitable hormonal milieu should be optimized for each condition by trial and error. In this respect, very valuable information can be obtained from animal models of the respective pathology.

Osteoarthritis is a chronic degenerative joint condition that used to be regarded as non-inflammatory. However, in recent decades, it has been associated with inflammation. Local inflammation affecting the synovial membrane and the infrapatellar fat pad in knee osteoarthritis is well known [8,9]. Recent research, however, also reveals systemic inflammation. Inflammatory biomarkers such as C-reactive protein, IL-1α, IL-6, IL-18, and tumor necrosis factor (TNF)-α have been associated with the presence and severity of osteoarthritis [10,11,12]. Positive correlation between osteoarthritis and the systemic inflammatory response index has been found [13], and some clinical trials have found beneficial effects of anti-inflammatory treatment [14]. This raises the question about the potential impact of osteoarthritis on oogenesis. While the majority of patients with osteoarthritis are women [15,16], for most of them, this question is irrelevant because the condition is age-related and typically appears after the natural decline in female fertility. However, there is a subset of younger patients who develop osteoarthritis during reproductive age. This can be due to genetic factors, e.g., mutations in certain collagen genes [17,18] and regulatory genes important for skeletal development [19]. Even more important is the role of lifestyle. Early-onset osteoarthritis has been reported to affect women of reproductive age whose occupation involves repetitive movements and overload of joints. The risk is aggravated in activities where injuries are common, such as in athletes [20,21,22,23] and professional ballerinas [24,25,26], but is present even when the strain on joints is not compounded by trauma, e.g., in professional musicians who are predisposed to osteoarthritis of the hands [24]. Another risk factor is obesity [20,21,27], which could contribute both by overloading the joints and by creating a pro-inflammatory tissue environment. These data challenge the traditional concept of osteoarthritis as a “wear and tear” disease of advanced age, and require treatment strategies for reproductive-age patients, among whom the incidence of the condition shows an increasing trend [28].

The relationship between reproductive factors and osteoarthritis is poorly studied. Higher age at menarche and higher parity have been found to be associated with lower osteoarthritis risk, but the mechanism of this connection is unknown [29,30]. With regard to reproductive-age women whose occupation in sport or art puts them at higher risk of osteoarthritis, it can be supposed that engagement with their careers may lead to postponed birth and, hence, lower fertility even if the condition itself has little impact.

In inflammatory disorders affecting predominantly the reproductive system, such as endometriosis and polycystic ovary syndrome, treatment strategies have a double goal: to address the underlying problem and to restore fertility. Hence, therapeutic approaches include reproductive hormones or their analogs in an effort to create a microenvironment that down-regulates inflammation and supports normal oogenesis and ovulation [7,31]. Osteoarthritis has a less direct relation to the reproductive system, but its origin and course are nevertheless influenced by gonadal hormones. Regarding estradiol, data are still insufficient and controversial. There are reports that osteoarthritis is associated with estradiol deficiency, implying a protective role of endogenous estradiol, and that estrogen replacement in ovariectomized animals and postmenopausal women results in improvement, at least in early-stage osteoarthritis [32,33,34]. Estrogen supplementation reduces the incidence of joint pain [35]. Estrogen has been found to have anti-inflammatory and protective effects on chondrocytes and to upregulate their synthesis of proteoglycan and collagen, slowing down the course of the condition [36]. Studies on animal models suggest anti-senescence action on chondrocytes as a possible mechanism for the benefits provided by estradiol in osteoarthritis [37]. Other authors, however, have found that higher levels of estradiol, on its own [38,39] or relative to testosterone [40], are associated with increased risk and faster progression of osteoarthritis. There are also reports finding no association between estradiol and osteoarthritis [41].

This discrepancy in the published literature shows that more research is needed. In this respect, valuable information can be obtained from animal models, where the pathology and prospective treatment approaches can be studied with far fewer technical and ethical limitations than in humans. An adequate mouse model of osteoarthritis is based on intra-articular injection of collagenase, which induces the pathological process. The resulting collagenase-induced osteoarthritis (CIOA) is characterized by degradation of articular cartilage, sclerosis of subchondral bone, formation of osteophytes, synovitis, and synovial hyperplasia, which are also hallmarks of human osteoarthritis [42,43]. The pathological changes are characterized by cartilage and bone erosion and osteophyte formation [44], making this experimental osteoarthritis similar to the erosive human osteoarthritis (because of the erosive joint changes) but also having some features more typical for human nodular arthritis (the presence of osteophytes). Recent studies by our team and other authors on the influence of estradiol in this model have found a beneficial effect [45,46,47].

Reproductive system-related aspects complicate the situation of young patients with osteoarthritis. The current opinion is that they should receive the same treatment as older patients [20]. While this may be true with regard to the condition per se, there is an additional consideration in young patients, namely, the preservation of their fertility. The impact (if any) of osteoarthritis on the female reproductive potential is unknown. Our team has recently performed a study on oogenesis in the CIOA mouse model, and has found a decreased oocyte maturation rate, enlargement of the meiotic spindle, and frequently found disorganization of microtubules and/or changes in the microfilament organization and the actin cap [48]. Given the similarity of observed pathological changes in CIOA and in human osteoarthritis, it seems likely that some of these disturbances of oogenesis may also be present in affected reproductive-age women. Moreover, while estradiol is not currently used for the treatment of osteoarthritis except in clinical trials, some of these studies provide encouraging results, mitigating the disease symptoms and progression, raising the possibilities of making hormonal treatment standard in the foreseeable future. However, any potential therapy of osteoarthritis with reproductive hormones, e.g., estradiol supplementation, could affect oogenesis in an unpredictable manner. In addition, it is likely that some young women with osteoarthritis would resort to in vitro fertilization, including the standard ovarian stimulation with follicle-stimulating hormone (FSH). The administration of this exogenous hormone poses two questions: first, whether it could affect osteoarthritis (we have found it to aggravate the pathological changes in the CIOA mouse model [45], and second, whether it would have the desired effect on ovaries, or the outcome would be hindered by the underlying disease.

The lack of data in the published literature necessitates research on the influence of reproductive hormones on oogenesis in the presence of osteoarthritis, which should initially be performed in animal models. The aim of our study, which builds upon and expands our previous research [48] with more experimental groups, was to combine the modulation effects of estradiol and FSH administration with the osteoarthritis-related inflammatory systemic background on oogenesis, and to evaluate key oocyte parameters. We found that the exogenous reproductive hormones modulated the impact of CIOA, leading to specific patterns of defects in oocytes.

## 2. Materials and Methods

Female outbred ICR mice aged 8–10 weeks with experimental collagenase-induced osteoarthritis (CIOA) were provided by the Institute of Microbiology at the Bulgarian Academy of Sciences (BAS), after some of them had been administered various in vivo treatment regimens with estradiol (E2) and follicle-stimulating hormone (FSH) during the acute phase of CIOA (described below). At the established phase of the disease and its chronification (day 18 to 30), the animals with or without additional treatment by reproductive hormones were subjected to ovarian hormonal stimulation according to a standardized scheme, then euthanized, and the ovulated eggs were isolated from the oviducts for analysis.

### 2.1. Groups of Oocytes Based on the Treatment of Animals

The experimental model of osteoarthritis has been described before [46] and is therefore described here briefly: after intraperitoneal anesthesia with 50 mg/kg sodium phenobarbital, osteoarthritis was induced by intra-articular injection of 2 IU/µL collagenase (from *Clostridium histolyticum*, Sigma-Aldrich, Hamburg, Germany) into the tibiotalar joint, which was day 0 of CIOA. Control animals were injected with sterile PBS into the tibiotalar joint instead of collagenase. The stages of osteoarthritis were defined as: acute phase (day 0–5), active development of inflammation (day 5–18), and the actual phase of osteoarthritis with chronification (after day 18). Estradiol treatment (17β-estradiol, Sigma-Aldrich) was administered *per os* at a dose of 4 μg in 0.300 μL sesame oil (Sigma-Aldrich) and 60 mg hazelnut-cocoa cream (Nutella, Ferrero Co., London, UK) per mouse. It was applied in parallel with CIOA for 30 days. For FSH treatment, the hormone (FSH, Sigma-Aldrich), diluted in sterile PBS, was injected intraperitoneally into the animals at a dose of 2 IU/mouse/day (0.2 mL) for a 10-day period (before CIOA induction or during days 0–10 of CIOA). On day 30, i.e., in the established phase of CIOA (with or without hormonal treatment), we obtained the animals and started hormonal stimulation for induction of ovulation.

Oocytes from animals with CIOA and different regimens of reproductive hormones were divided into groups to assess the effects of E2 and FSH on key oocyte structures: actin and tubulin cytoskeleton, and chromatin. The groups of oocytes are described below.

**Control**: Oocytes from mice without CIOA of the same age as the other groups in this study.

**Control^Nut^**: Obtained from animals that were fed hazelnut-cocoa cream (Nutella) the same way as animals that obtained estradiol in this cream, but without CIOA or exogenous hormones. To account for the eventual effects of the hazelnut-cocoa cream, the estradiol-treated groups needed such a separate control group receiving the same cream but without the hormone.

**CIOA**: Oocytes obtained from females with CIOA. Induction of CIOA started at 1 month of age, and ovarian stimulation started on day 30.

**CIOA^Nut^**: Oocytes from animals with CIOA that were given in parallel Nutella for 30 days starting at 1 month of age.

**E2^Nut^**: Oocytes from mice treated for 30 days with estradiol (E2) dissolved in hazelnut-cocoa cream Nutella (starting at 1 month of age);

**CIOA + E2^Nut^**: Oocytes obtained from animals with CIOA for 30 days and parallelly treated with E2 (in Nutella) for the same period.

**10 FSH**: Oocytes from animals without CIOA treated with FSH for 10 days (starting at 1 month of age). The ovarian stimulation for oocyte retrieval was performed after the 30th day from the start of the FSH treatment.

**CIOA^10 FSH^**: Oocytes obtained from animals with CIOA for 30 days, treated with FSH during the first 10 days after the induction of arthritis.

**10 FSH + CIOA**: Oocytes from animals treated with FSH for 10 days followed by induction of arthritis for 30 days.

The groups related to studying the effects of estradiol (Control^Nut^, CIOA^Nut^, E2^Nut^ and CIOA + E2^Nut^) included 7 animals each, while those related to studying FSH (Control, CIOA, 10 FSH, CIOA^10FSH^, and 10 FSH + CIOA) included 10 animals each; the larger group size was intended to allow distinction between the results for the groups CIOA^10FSH^ and 10 FSH + CIOA, which both combined FSH and CIOA and differed only in the time course.

A graphical presentation of treatment regimens for the above-described groups is shown in Supplementary Table S1.

### 2.2. Hormonal Ovarian Stimulation, Isolation, and Fixation of Oocytes

After the above-described treatments, work with the animals was performed at the Department of Biology, Medical University of Sofia. They were subjected to ovarian hormonal stimulation to obtain synchronously ovulated oocytes. The hormonal doses were adjusted to the relatively large weight of the mice in our study due to their fully adult age. The females were injected intraperitoneally with 12 IU FSH combined with luteinizing hormone (Meriofert, IBSA Farmaceutici, Milan, Italy) to induce multiple ovulation, and 48 h later, with 14 IU human chorionic gonadotropin (Choriomon, IBSA Farmaceutici, Italy) for synchronous ovulation of the oocytes.

All animals were anesthetized and euthanized 16 h later. Their oviducts were dissected and manipulated in Leibowitz medium (Sigma-Aldrich, Germany) with 0.3% bovine serum albumin (BSA; Sigma-Aldrich, Germany) on a warming plate at 37 °C (Labotect, Gottingen, Germany). Cumulus-oocyte complexes were collected from the dissected oviduct ampullae, then washed in clean Leibowitz medium and treated with 0.5 mg/mL hyaluronidase (Sigma-Aldrich, Germany) to remove the cumulus layer. Denuded oocytes were washed in PBS-BSA buffer (PBS, pH 7.2, with 0.3% BSA). All these steps were carried out in sterile embryological Petri dishes (Corning, Corning, NY, USA) under a stereomicroscope (Zeiss, Oberkochen, Germany).

The oocytes were transferred to sterile 96-well round-bottom plates (Corning, USA), fixed with 2% paraformaldehyde in permeabilization buffer (PBS with 0.02% Triton X-100) for 45 min at 37 °C. Then they were washed twice (10 min each) and stored at 4 °C for 24 h in 0.02% sodium azide supplemented PBS-BSA buffer.

### 2.3. Immunofluorescent Staining of the Oocytes

The oocytes were incubated for 45 min at 37 °C with a monoclonal mouse anti-α-tubulin antibody (clone DM1A, Sigma-Aldrich), diluted 1:1000 in dilution buffer (PBS, pH 7.2, with 0.3% BSA and 1% sodium azide) and washed twice for 10 min each with washing buffer (PBS, pH 7.2, with 0.3% BSA and 1 μL/mL Tween 20). This was followed by incubation (45 min at 37 °C) with FITC-labeled anti-mouse IgG antibody (Sigma-Aldrich) for microtubule visualization, diluted 1:200 in dilution buffer, together with phalloidin-TRITC (Sigma-Aldrich, working concentration 1 μg/mL) for fibrillar actin visualization and Hoechst (Hoechst 33258 at a working concentration of 8 μg/mL, Sigma-Aldrich) for chromatin visualization. After fluorescent staining, the oocytes were washed twice for 10 min each in washing buffer and incubated in polyvinyl alcohol (Fluka, Darmstadt, Germany) with ascending concentrations of 10%, 30%, and 50%. The oocytes were mounted on glass slides in a drop of 20 µL of 100% polyvinyl alcohol and covered with a coverslip to be ready for microscopy.

Observation and documentation were performed on an epifluorescence microscope (Axioskop 20, Zeiss, Gottingen, Germany) as well as on a laser scanning confocal microscope (LSCM, Leica TCS SPE, Leica, Wetzlar, Germany).

### 2.4. Epifluorescence and Laser Scanning Confocal Microscopy of the Oocytes

Evaluation of the microtubule and actin cytoskeleton, the chromatin status, and the maturation stage of the oocytes was performed by epifluorescence microscopy and digital image capture. Some of the oocytes were selected for laser scanning confocal microscopy (LSCM at 0.1 μm optical section). The measurement of the visualized structures in the oocytes was performed with the program LAS AF (Leica Application Suite Advanced Fluorescence, Version 1.8.2). Using the LSCM results as standards, the structures from epifluorescence microscopy were measured, and all morphometric data were recorded: length and width of the meiotic spindle and width of its poles, as well as size of the actin cap. The mean values plus or minus a standard deviation for controls were considered normal. In addition to these measurements, the tubulin cytoskeleton was also characterized by the presence or absence of tubulin asters in the cytoplasm and at spindle poles, and by spindle integrity, i.e., wholly organized meiotic spindle, overall normal spindle with single detached microtubules, or totally disorganized spindle. For the microfilament cytoskeleton, the presence of an actin cap and/or halo was assessed, as well as the size of the cap, with the same criterion for normal, small, and large size as with the spindle. For the chromatin status, metaphase plates were characterized by chromosome alignment (aligned, misaligned, or outside the spindle) or a degenerated state.

### 2.5. Statistical Analysis

The statistical analysis of the results was performed using the IBM SPSS Statistics for Windows program (Version 27.0/28.0/29.0, IBM Corporation, Armonk, NY, USA).

Non-parametric tests: Fisher–Freeman–Halton Exact Test for independence was used to compare categorical data.

When performing multiple comparisons, the Hochberg “p*_Hoch_*” correction [49] was applied to the statistical significance result “p*_i_*” from the Fisher–Freeman–Halton Exact Test. The procedure consists of a sequentially rejective algorithm that creates a ranking of “p*_i_*” results and adjusts “p*_i_*” to “p*_Hoch_*”. Values of *p* ≤ 0.05 were considered statistically significant.

## 3. Results

The study included 1370 ovulated mouse oocytes subdivided into groups according to the treatment (controls, CIOA, and the groups treated by estradiol or FSH). Their maturation status is shown in Table 1 and Table 2: mainly M I and M II, as well as extremely immature GV or GVBD (before M I), some transitional anaphase I to telophase stages (Ana I/Telo I), unusual findings of anaphase II to telophase II (Ana II/Telo II), or degenerative appearance.

### 3.1. Morphological Peculiarities of the Oocytes from the Different Groups

**Figure 1:**
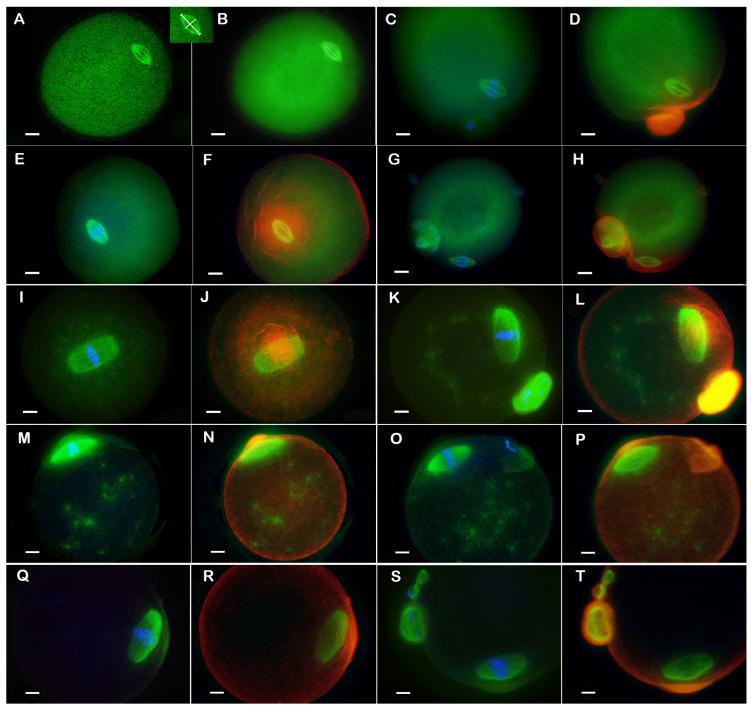
Microscopic images of Control, Control^Nut^, CIOA^Nut^, E2^Nut^, and CIOA + E2^Nut^ mouse oocytes. (**A**–**D**): Controls. (**A**)—LSCM image (FITC, green) of an M I oocyte showing the precise measurements of the spindle (inset)—length 21 µm, width 10 µm, and pole width 2 µm. (**B**)—Epifluorescent image of the same oocyte and its spindle. Based on such pairs of photos, the selected confocal images were used to adjust measurements of all epifluorescence images. (**C**)—Epifluorescence of M II meiotic spindle (FITC, green) with well-aligned metaphase plate (blue) and chromatin of the polar body (bottom). (**D**)—The same M II oocyte with its spindle (green), actin cap (red), and its polar body. Normal spindle size, appearance, metaphase plate, and actin cap are seen. (**E**–**H**): Control^Nut^ oocytes. (**E**,**F**)—M I Control^Nut^ oocyte, (**G**,**H**)—M II Control^Nut^ oocyte. Oocyte structures (spindle, metaphase, and actin cap) are normal. (**I**–**L**): CIOA^Nut^ oocytes. (**I**,**J**)—M I CIOA^Nut^, (**K**,**L**)—M II CIOA^Nut^. Large and wide spindles with broad poles and cytoplasmic tubulin asters are seen; metaphase plates are aligned. (**M**–**T**): Oocytes of the estradiol-treated groups. (**M**–**P**): E2^Nut^. (**M**,**N**)—M I of E2^Nut^ oocyte, (**O**,**P**)—M II of E2^Nut^ oocyte. The large spindles are accompanied by ooplasmic tubulin asters; a good metaphase plate is seen in both cells. (**Q**–**T**): CIOA + E2^Nut^. (**Q**,**R**)—M I of CIOA + E2^Nut^, (**S**,**T**)—M II of CIOA + E2^Nut^. The cells show large spindles with wide poles, aligned chromosomes, and large actin caps. Images (**C**,**E**,**G**,**I**,**K**,**M**,**O**,**Q**,**S**) show spindle (FITC, green) and metaphase plate (Hoechst 33258, blue), images (**D**,**F**,**H**,**J**,**L**,**N**,**P**,**R**,**T**)—spindle (FITC, green) and actin (TRITC, red). Original magnification 1000×, bar = 10 µm.

**Control** group: These oocytes showed a normal spindle with dimensions corresponding to those reported in the literature [50], and good quality of chromosomes aligned at its equator. The actin cytoskeleton was presented by an actin cap, the polar structure anchoring the spindle in the oocyte periphery. The actin caps of the Control group were of normal position, morphology, and size. Laser scanning confocal microscopy imaging was used for the adjustment of measurements. The average parameters for M I were: spindle length 26.26 µm, spindle width 11.64 µm, spindle pole width 3.6 µm, and actin cap size 63.40 µm. For M II, the mean spindle length was 24.71 µm, the spindle width 12.64 µm, the poles were 3.36 µm wide, and the actin cap was 62.40 µm. LSCM and epifluorescent images are compared in Figure 1A–D. The proportion of mature M II stage oocytes was over 56%, which was the highest among all of the studied groups. The number of extremely immature and degenerated oocytes had the lowest value among the groups of oocytes. This Control group was used for comparison with the groups CIOA and CIOA/FSH (10 FSH, 10 FSH + CIOA and CIOA^10 FSH^).

**Figure 2:**
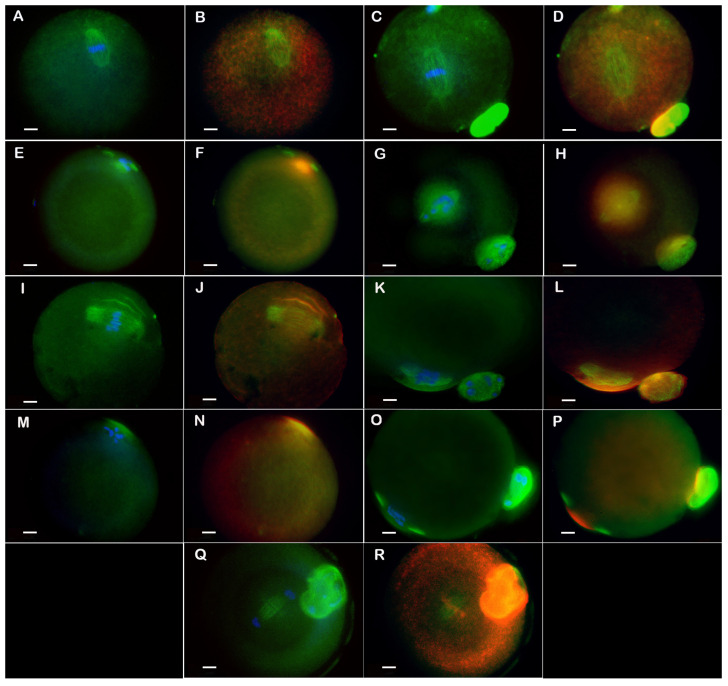
Oocytes of CIOA, 10 FSH, CIOA^10 FSH^ and 10 FSH + CIOA groups. (**A**–**D**): CIOA oocytes. (**A**)—M I of CIOA oocyte, (**C**)—M II of CIOA oocyte. Meiotic spindles of a larger and wider type and with wide poles. The metaphase plates are of good quality and properly aligned. (**B**,**D**)—the same CIOA oocytes showing an actin halo replacing their actin cap. (**E**–**H**): 10 FSH oocytes. (**E**)—M I of 10 FSH oocyte, (**G**)—M II of 10 FSH oocyte. The spindles are elongated and slender with focused poles, but a cloud of depolymerized tubulin surrounds the misaligned chromosomes. (**F**,**H**)—the same oocytes showing co-localization of their actin with the cloud of tubulin around the chromosomes. (**I**–**P**): Oocytes of groups combining CIOA and FSH treatment. (**I**–**L**): CIOA^10 FSH^ oocytes. (**I**,**J**)—M I of CIOA^10 FSH^ group, (**K**,**L**)—M II of CIOA^10 FSH^ group. The large meiotic spindles with relatively well-arranged metaphases are surrounded by actin caps. (**M**–**P**): 10 FSH + CIOA oocytes. (**M**,**N**)—M I of 10 FSH + CIOA: the spindle is disorganized, the chromosomes are scattered and an actin halo is seen. (**O**,**P**)—M II oocyte of 10 FSH + CIOA group: the spindle is long and slender with tight poles, the chromosomes are misaligned and not attached to the spindle, and the actin cap is small. (**Q**,**R**): Post-MII stage (Telophase II) in an oocyte from the 10 FSH group. (**Q**) The chromatin forms two masses between which a parallel array of microtubules is seen. (**R**) At the equator, actin is concentrated in a contractile ring. Next to the spindle is the polar body. (**A**,**C**,**E**,**G**,**I**,**K**,**M**,**O**,**Q**) show spindle (FITC, green) and metaphase plate (Hoechst 33258, blue), images (**B**,**D**,**F**,**H**,**J**,**L**,**N**,**P**,**R**)—spindle (FITC, green) and actin (TRITC, red). Original magnification 1000×, bar = 10 µm.

**Control^Nut^** group: These oocytes also had normal-sized meiotic spindles, well-aligned metaphase chromosomes within them, and normal actin caps. Epifluorescent images for this group are shown in Figure 2A–D. The distribution of meiotic maturation stages was comparable to the Control group with no statistically significant difference. The spindle size and morphology were normal (mean length 20.27 µm for M I and 20.05 µm for M II, mean width 11.55 µm for M I and 12.89 µm for M II, poles 3.03 µm and 3.17 µm wide for M I and M II, respectively), the actin caps also had normal size (mean 69.83 µm for M I and 66.00 µm for M II) and appearance, and the metaphase plates were well aligned. The morphological parameters showed no statistically significant differences in comparison to the Control group. The Control^Nut^ group was the control of groups E2^Nut^, CIOA^Nut^, and CIOA + E2^Nut^.

**CIOA^Nut^**. The group was distinguished by a high percentage of cells with long and wide spindles (mean length 38.26 µm for M I and 41.27 µm for M II, mean width 19.30 µm for M I and 19.09 µm for M II) with wide poles (6.17 µm and 5.96 µm mean width for M I and M II, respectively) and additional tubulin stars (asters) in the cytoplasm (see Figure 1I for M I oocytes, and Figure 1K for M II). However, the spindle had a normal overall appearance, and this corresponded to the correct alignment of chromosomes in the metaphase plate. The actin caps, similarly to the spindles, were relatively large (mean size 70.98 for M I and 69.65 for M II), as shown in Figure 1L. In many cells, a non-polarized actin halo was accompanying or had replaced the actin cap and was visible as a ring-shaped cloud (see Figure 1J).

**E2^Nut^** group oocytes are shown in Figure 1M–P. Meiotic spindles of this group were shorter and slimmer (mean length 37.62 µm for M I, 35.82 µm for M II, and mean width 15.91 µm for M I, 18.33 µm for M II), with focused poles (3.23 µm and 4.93 µm for M I and M II, respectively). Tubulin asters were frequently observed in the cytoplasm (in over 70% of the cells). The chromosomes were well aligned. Actin caps were small (51.31 µm for M I and 54.19 µm for M II), and an actin halo was not observed in the group.

**CIOA + E2^Nut^**. Oocytes of this group are shown in Figure 1Q–T. They were distinguished by a low percentage of meiotically mature oocytes (21.82%) and the highest percentage of degenerated oocytes (25.45%). The spindles were large (mean size 46.90 µm by 19.29 µm for M I and 36.80 µm by 19.02 µm for M II) and with the widest poles of all groups (mean 7.85 µm in M I and 7.43 µm for M II spindles). The alignment of chromosomes was predominantly normal. An actin halo was present in 33.33% of the M I of this group. The M II oocytes had their actin caps well-formed but of larger size (7.63 µm), similar to the large spindles.

**CIOA** group oocytes are shown in Figure 2A–D. In this group, the proportion of meiotically mature oocytes was lower (26.42% M II) than in the Control with which this group was compared (56.06% M II). Characteristic features of the group were the large spindles: both longer (mean 37.59 µm for M I and 37.04 µm for M II) and wider (mean width 18.04 µm for M I and 17.34 µm for M II), with wide poles (mean 7 µm for M I and 6.81 µm for M II) compared to the Control oocytes. In addition, actin caps were often absent (in 61.87% of MI and 52.94% of M II), being replaced by a non-polarized actin halo, seen as a ring-shaped cloud (Figure 2B,D). Despite these characteristic cytoskeletal abnormalities, the chromosomes showed good quality and alignment in metaphase plates.

**10 FSH**: The group was characterized by slender (mean 13.13 µm for M I and 13.20 µm for M II), elongated spindles (mean 38.41 µm for M I and 36.59 µm for M II) with tighter and focused poles (mean 5.32 µm for M I and 4.71 µm for M II), as well as small actin caps (mean 41.44 µm for M I and 40.03 µm for M II), see Figure 2E–H. A “cloud” of depolymerized tubulin was observed in the central region of the spindle, around the chromosomes. This was accompanied by unaligned chromosomes. There was also a higher percentage of disorganized spindles and degenerated chromatin compared to the other groups.

**CIOA^10 FSH^**. The oocytes had larger spindles in comparison to the other FSH groups, but their mean morphometric values were generally closer to the other CIOA groups (see Figure 2I,J for M I as well as Figure 2K,L for M II). The meiotic spindles were longer (mean 40.92 µm for M I and 41.62 µm for M II) but of comparable width (17.57 µm for M I and 16.41 µm for M II) and tighter poles (5.72 µm for M I and 5.73 µm for M II) than CIOA. A tubulin cloud was present in about 17 to 19% of oocytes, chromosome misalignment was seen in more than 30% of cells, and the actin caps remained small (typical for the FSH groups).

**10 FSH + CIOA** group oocytes are shown in Figure 2M,N for M I, and Figure 2O,P for M II. Meiotic spindles were the slenderest (11.78 µm for M I and 10.98 µm for M II) with the most focused poles (4.50 µm for M I and 4.29 µm for M II) compared to the other FSH groups. This group had the highest proportion of oocytes with disorganized spindles, tubulin clouds, misaligned metaphase plates, degenerated chromatin, and actin halo. The group also showed the smallest mean width of actin caps.

A characteristic finding for FSH-treated groups (10 FSH, CIOA^10FSH^, and 10 FSH + CIOA) was unusual stages between MI and MII or post-MII. They included oocytes in Ana I/Telo I transition, rarely seen in other groups, as well as Ana II/Telo II oocytes, not observed in other groups at all. The highest percentage of these stages was registered for 10 FSH and 10 FSH + CIOA groups: Ana I/Telo I transition was registered in 10.75% of 10 FSH and 9.44% of 10 FSH + CIOA oocytes, and post-MII (Ana II/Telo II) stages were seen in 7.48% of 10 FSH and 8.39% of 10 FSH + CIOA oocytes, as it was shown in Table 2. Figure 2Q, R shows a representative cell. For comparison, the percentage of Ana I/Telo I oocytes in non-FSH groups was 4.94% or lower, and not a single Ana II/Telo II cell was observed in these groups.

### 3.2. Data Analysis for Oocytes from CIOA and/or Estradiol Groups

In the groups of oocytes Control^Nut^, CIOA^Nut^, E2^Nut^ and CIOA + E2^Nut^, the Control^Nut^, as expected, had the best maturation rate of almost 53% (Table 1). This rate was decreased in the other three groups, and the lowest maturation rate was found in CIOA + E2^Nut^, which was influenced by both factors. The same group showed the highest proportion of degenerated oocytes (over 25%) and of pre-M I oocytes (over 30%).

The oocyte cytoskeleton was analyzed for tubulin and actin parameters. The microtubule peculiarities were subdivided into spindle size (length and width), pole width, spindle integrity, as well as the presence of tubulin asters. The actin cytoskeletal parameters included the size of the actin cap and the presence of a non-polar actin halo in the cytoplasm. The chromatin of the cells was analyzed according to its alignment and quality.

**Figure 3:**
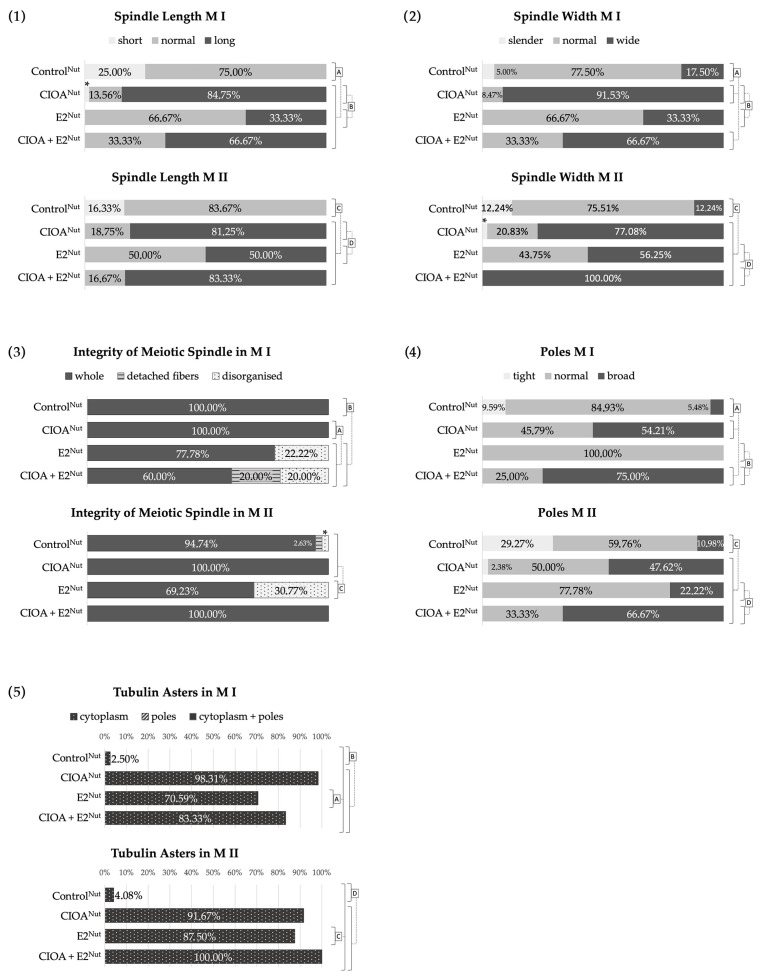
Comparison of the meiotic spindle parameters in groups subjected to osteoarthritis and/or E2. (**1**). Spindle length. Categories: for M I—short: <18.74 µm; normal: 26.26 ± 7.52 µm; long: >33.77 µm; for M II—short: <17.25 µm; normal: 24.71 ± 7.46 µm; long: >32.17 µm. (**Top**): Spindle length in M I. A—comparison of Control^Nut^ versus CIOA^Nut^, E2^Nut^, and CIOA + E2^Nut^ (for all: p*_i_* < 0.01; p*_Hoch_* < 0.01). B—comparison between CIOA^Nut^ and E2^Nut^ (p*_i_* < 0.01; p*_Hoch_* < 0.01). * CIOA^Nut^—1.69% of oocytes with a short spindle in M I. (**Bottom**): Spindle length in M II. C—comparison of Control^Nut^ versus CIOA^Nut^, E2^Nut^, and CIOA + E2^Nut^ (for all: p*_i_* < 0.01; p*_Hoch_* < 0.01). D—comparison between CIOA^Nut^ and E2^Nut^ (p*_i_* < 0.01; p*_Hoch_* < 0.05). (**2**). Spindle width. Categories for M I—slender: <9.13 µm; normal: 11.64 ± 2.51 µm; wide: >14.15 µm. Categories for M II—slender: <9.96 µm; normal: 12.64 ± 2.68 µm; wide: >15.32 µm. (**Top**) M I: A—Control^Nut^ versus CIOA^Nut^ and CIOA + E2^Nut^ (p*_i_* < 0.01; p*_Hoch_* < 0.05). B—comparison between CIOA^Nut^ and E2^Nut^ (p*_i_* < 0.01; p*_Hoch_* < 0.01). (**Bottom**) M II: C—comparison of Control^Nut^ vs. CIOA^Nut^, E2^Nut^, and CIOA + E2^Nut^ (p*_i_* < 0.01; p*_Hoch_* < 0.01). D—comparison between E2^Nut^ and CIOA + E2^Nut^ (p*_i_* < 0.01; p*_Hoch_* < 0.01). * CIOA^Nut^—2.08% of oocytes with a slender spindle in M II. (**3**). Spindle integrity. (**Top**) M I: A—comparison of CIOA^Nut^ versus each of the other groups without Control^Nut^ (for all: p*_i_* < 0.01; p*_Hoch_* < 0.05). B—comparison of Control^Nut^ versus E2^Nut^ and CIOA + E2^Nut^ (for all: p*_i_* < 0.01; p*_Hoch_* < 0.05). (**Bottom**) M II: C—comparison of E2^Nut^ versus Control^Nut^ and CIOA^Nut^ (p*_i_* < 0.01; p*_Hoch_* < 0.05). * Control^Nut^—2.63% oocytes with a disorganized spindle in M II. (**4**). Spindle pole width. Categories for M I—tight: <1.70 µm; normal: 3.60 ± 1.90 µm; broad: >5.50 µm. Categories for M II—tight: <1.59 µm; normal: 3.56 ± 1.97 µm; broad: >5.53 µm. (**Top**): data for M I. A—comparison of Control^Nut^ versus CIOA^Nut^ and CIOA + E2^Nut^ (p*_i_* < 0.01; p*_Hoch_* < 0.01). B—comparison between E2^Nut^ and CIOA + E2^Nut^ (p*_i_* < 0.01; p*_Hoch_* < 0.01). (**Bottom**): data for M II. C—comparison of Control^Nut^ vs. CIOA^Nut^, E2^Nut^ and CIOA + E2^Nut^ (p*_i_* < 0.01; p*_Hoch_* < 0.01). D—comparison between E2^Nut^ and CIOA + E2^Nut^ (p*_i_* < 0.01; p*_Hoch_* < 0.01). (**5**). Presence of tubulin asters in the ooplasm. (**Top**) M I: A—comparison Control^Nut^ versus CIOA^Nut^, E2^Nut^ and CIOA + E2^Nut^ (for all p*_i_* < 0.001; p*_Hoch_* < 0.001). B—comparison of E2^Nut^ versus Control^Nut^ on the one hand, and versus CIOA^Nut^ and CIOA + E2^Nut^ on the other (for all p*_i_* < 0.001; p*_Hoch_* < 0.001). (**Bottom**) M II: C—comparison Control^Nut^ versus CIOA^Nut^, E2^Nut^, and CIOA + E2^Nut^ (for all p*_i_* < 0.001; p*_Hoch_* < 0.001). D—comparison of E2^Nut^ versus Control^Nut^ on the one hand, and versus CIOA^Nut^ and CIOA + E2^Nut^ on the other (for all p*_i_* < 0.001; p*_Hoch_* < 0.001). In all graphs, p*_i_*—Fisher–Freeman–Halton exact test; p*_Hoch_*—correction of p*_i_* for multiple comparisons according to the Hochberg method.

The results concerning the spindle showed predominantly normal spindle morphology and size (length and width) with normal poles for the Control^Nut^ group of oocytes. This group generally had smaller spindles in both M I and M II, while the other three groups had almost no short spindles and a significantly higher percentage of long spindles for both M I and M II, especially CIOA^Nut^ and CIOA + E2^Nut^ groups (Figure 3(1)). The E2^Nut^ oocytes had more normal and fewer large spindles, while CIOA^Nut^ and CIOA + E2^Nut^ had over 2/3 large spindles for both M I and M II. The spindle length differed significantly between Control^Nut^, on the one hand, and all treated groups, on the other, and also between CIOA^Nut^ and E2^Nut^ (Figure 3(1)).

The spindle width data (Figure 3(2)) and the pole width data (Figure 3(4)) showed the same tendency as spindle length: the more long and wide spindles a particular group contained, the more broad poles were observed in it. It should be noted, however, that the proportion of spindles with broad poles was lower than the proportion of long and wide spindles. This was especially true for group E2^Nut^.

The overall characteristics concerning morphologically normal microtubular organization without detached fibers (whole), spindles with some detached microtubules, or fully disorganized tubulin structure of the spindle are shown in Figure 3(3). The data showed normal spindle integrity for oocytes from the Control^Nut^, CIOA^Nut^, and M II of the CIOA + E2^Nut^. Both M I and M II oocytes of the E2^Nut^ group showed a significant percentage of disorganized spindles in comparison with the other three groups. The M I oocytes from the group CIOA + E2^Nut^ showed both spindles with detached microtubules and disorganized spindles (20% each). These spindle abnormalities were extremely low in the Control^Nut^ and not found in the CIOA^Nut^ oocytes. Statistically significant differences were registered between Control^Nut^ and E2^Nut^ in both M I and M II, while CIOA^Nut^ resembled Control^Nut^ for this parameter.

The oocyte groups influenced by estradiol and/or CIOA (E2^Nut^, CIOA^Nut^ and CIOA + E2^Nut^) were characterized by tubulin asters in the ooplasm (data in Figure 3(5), and photos in Figure 1I–L and Figure 2M–P). This specific peculiarity distinguished E2^Nut^, CIOA^Nut^, and CIOA + E2^Nut^ groups from all of the other groups in the current study, including the FSH-treated groups. Data from the groups subjected to only one of the two factors (E2^Nut^ or CIOA^Nut^) showed that the aster-inducing influence of arthritis was stronger than that of estradiol, and this difference was significant.

**Figure 4:**
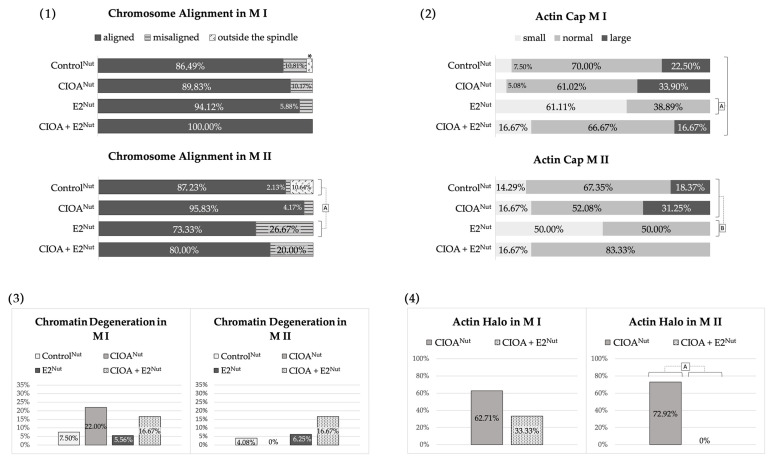
Chromatin and actin parameters in groups influenced by osteoarthritis and/or E2. (**1**). Chromosome alignment. (**Top**): M I. * Control^Nut^—2.70% of oocytes with chromosomes that were outside the spindle. (**Bottom**): M II. A—comparison of E2^Nut^ versus Control^Nut^ (p*_i_* < 0.01; p*_Hoch_* < 0.05). (**2**). Distribution of actin cap size. Categories for M I—small: <49.86 µm; normal: 63.40 ± 13.54 µm; large: >76.94 µm. Categories for M II—small: <49.68 µm; normal: 62.40 ± 12.72 µm; large: >75.12 µm. (**Top**): M I. A—comparison of E2^Nut^ versus Control^Nut^, CIOA^Nut^, and CIOA + E2^Nut^ (for all: p*_i_* < 0.01; p*_Hoch_* < 0.05). (**Bottom**): M II. B—comparison of E2^Nut^ versus Control^Nut^ and CIOA^Nut^ (for all: p*_i_* < 0.01; p*_Hoch_* < 0.01). (**3**). Presence of degenerated chromatin. (**Left**): M I. (**Right**): M II. A significant difference was obtained between the groups CIOA^Nut^ and CIOA + E2^Nut^ (M I and M II: p*_i_* < 0.05). These two groups showed no statistically significant differences in these categories. (**4**). Presence of actin halo. (**Left**): M I. (**Right**): M II. A—comparison between CIOA^Nut^ and CIOA + E2^Nut^ (for all: p*_i_* < 0.001; p*_Hoch_* < 0.001). In all graphs, p*_i_*—Fisher–Freeman–Halton exact test; p*_Hoch_*—correction of p*_i_* for multiple comparisons according to the Hochberg method.

The metaphase plates of groups Control^Nut^, E2^Nut^, CIOA^Nut^, and CIOA+E2^Nut^ were classified as well-aligned, misaligned but in the middle region of the meiotic spindle, or misaligned and scattered out of the spindle (data shown in Figure 4(1)). The percentages of cells with degenerative and fuzzy chromosome appearance are given in Figure 4(3). Both figures show that the majority of oocytes from the above groups had metaphase plates of good quality and properly aligned in the meiotic spindles: more than 86% for M I and higher than 73% for M II for all four groups. Curiously, the best results for this characteristic were observed for M I of CIOA + E2^Nut^ group.

Chromatin degeneration levels in Control^Nut^, E2^Nut^, CIOA^Nut^, and CIOA + E2^Nut^ groups were also relatively low. Cells with degenerated chromatin were found mainly in CIOA^Nut^ (only in M I) and in CIOA + E2^Nut^ (M I and M II in equal proportion). It should be noted that this chromatin degeneration was observed despite the high proportion of good metaphase alignment for these two groups.

Microfilament organization in the oocytes from groups Control^Nut^, E2^Nut^, CIOA^Nut^, and CIOA + E2^Nut^ displayed an actin cap, as expected, in more than 90% of metaphase oocytes. In many cells from these groups, however, another finding was also observed: a diffuse microfilament halo (data in Figure 4(2,4), photos in Figure 1J,L).

Differences in the size of the actin cap were observed between the groups. As shown in Figure 4(2), compared to Control^Nut^, actin caps in CIOA^Nut^ had a tendency to be slightly larger, while those in E2^Nut^ tended to be smaller, the only one of these differences that reached statistical significance. In the “combined influence” CIOA + E2^Nut^ group, caps were on average slightly smaller than in Control^Nut^ but with overall similar distribution and predominantly normal size, as if the “enlarging” effect in CIOA^Nut^ and the diminishing effect in E2^Nut^ had neutralized each other. These patterns were observed for both M I and M II.

The actin halo was often present in CIOA^Nut^ M I and M II oocytes, and in CIOA + E2^Nut^ M I oocytes. There was a statistically significant difference between the two groups in M II. The halo was not observed in the other groups (Figure 4(4)).

In summary, the groups for the study of the influence of osteoarthritis and/or estradiol combined with Nutella (Control^Nut^, E2^Nut^, CIOA^Nut^, and CIOA + E2^Nut^) displayed specific phenotypes if CIOA and E2 effects were assessed alone (CIOA^Nut^ and E2^Nut^ compared to the Control^Nut^). CIOA was associated with a decrease in the maturation rate, enlargement of the spindle (length, width and poles), formation of an actin halo, and chromatin degeneration restricted to M I. Estradiol alone also led to maturation rate decrease (even stronger than in CIOA^Nut^) and spindle enlargement (though not so much as osteoarthritis) but had the opposite influence on the actin cap, which was often small. All three affected groups (E2^Nut^, CIOA^Nut^, and CIOA + E2^Nut^) had ooplasmic tubulin asters and generally well-aligned metaphase plates in the spindle equator. When both factors influenced the oogenesis (CIOA + E2^Nut^), the specific effects of osteoarthritis and estradiol were “summed up”, which, with regard to the actin cap, led to an average appearance. However, this group had the worst maturation rate and the highest percentage of degenerated cells; thus, the fact that so few cells made it to M II was more important than any normal values of individual parameters.

### 3.3. Data Analysis for Oocytes from CIOA and/or FSH Groups

In the groups of oocytes for study of the effects of osteoarthritis and/or FSH (Control, CIOA, 10 FSH, CIOA^10 FSH^, and 10 FSH + CIOA), the Control, as expected, had the best maturation rate of over 56% M II oocytes (Table 2). This rate was decreased in the other four groups, with the lowest maturation rate for the CIOA group. The same group showed the highest proportion of M I oocytes (over 72%). Moreover, as mentioned before, the FSH-treated groups were distinguished by a relatively high percentage of transitional Ana I/Telo I oocytes (rare in other groups) and of unique post-M II (Ana II/Telo II) oocytes not observed in any of the other groups.

The oocyte characteristics that were studied were, again, parameters of the tubulin cytoskeleton (spindle length, width, pole width and integrity, as well as presence of tubulin asters), microfilament structures (actin cap size and the eventual presence of actin halo), and the chromatin status.

**Figure 5:**
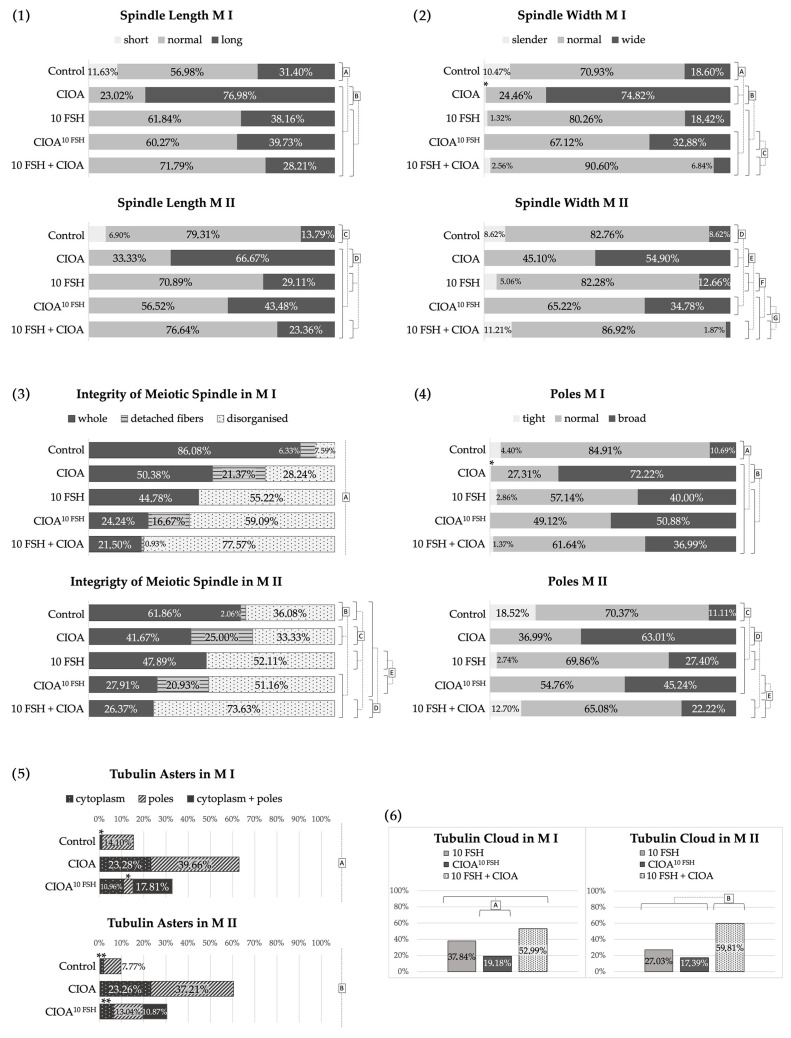
Comparison of the meiotic spindle parameters in groups subjected to osteoarthritis and/or FSH. (**1**). Spindle length. Categories for M I—short: <18.74 µm; normal: 26.26 ± 7.52 µm; long: >33.77 µm. Categories for M II—short: <17.25 µm; normal: 24.71 ± 7.46 µm; long: >32.17 µm. (**Top**): Data for meiotic spindle in M I. A—comparison of Control versus each of the other groups (for all comparisons: p*_i_* < 0.01; p*_Hoch_* < 0.05). B—comparison of CIOA versus 10 FSH, CIOA^10 FSH^ and 10 FSH + CIOA (for all: p*_i_* < 0.01; p*_Hoch_* < 0.01). (**Bottom**): Data for meiotic spindle in M II. C—comparison of Control versus each of the groups (for all comparisons: p*_i_* < 0.01; p*_Hoch_* < 0.05). D—comparison of CIOA versus 10 FSH and 10 FSH + CIOA (for all comparisons: p*_i_* < 0.01; p*_Hoch_* < 0.01). (**2**). Spindle width. Categories for M I—slender: <9.13 µm; normal: 11.64 ± 2.51 µm; wide: >14.15 µm. Categories for M II—slender: <9.96 µm; normal: 12.64 ± 2.68 µm; wide: >15.32 µm. (**Top**): Data for M I. A—comparison of Control versus CIOA and CIOA10 FSH and 10 FSH + CIOA (for all comparisons: p*_i_* < 0.01; p*_Hoch_* < 0.05). B—comparison of CIOA versus 10 FSH, CIOA^10 FSH^ and 10 FSH + CIOA (for all: p*_i_* < 0.01; p*_Hoch_* < 0.01). C—comparison of CIOA^10 FSH^ versus 10 FSH + CIOA (p*_i_* < 0.01; p*_Hoch_* < 0.01). * CIOA—0.72% oocytes with a slender spindle in M I. (**Bottom**): Data for M II. D—comparison of Control versus CIOA and CIOA^10 FSH^ (for all comparisons: p*_i_* < 0.01; p*_Hoch_* < 0.01). E—comparison of CIOA versus 10 FSH and 10 FSH + CIOA (for all comparisons: p*_i_* < 0.01; p*_Hoch_* < 0.01). F—comparison of 10 FSH versus CIOA^10 FSH^ and 10 FSH + CIOA (for all: p*_i_* < 0.01; p*_Hoch_* < 0.05). G—comparison of CIOA^10 FSH^ versus 10 FSH + CIOA (p*_i_* < 0.01; p*_Hoch_* < 0.01). (**3**). Spindle integrity. (**Top**): Data for M I. A—there is a statistically significant difference between each group compared to the other (all: p*_i_* < 0.01; p*_Hoch_* < 0.01). (**Bottom**): Data for M II. B—comparison of Control compared to CIOA, CIOA^10 FSH^ and 10 FSH + CIOA (all: p*_i_* < 0.05; p*_Hoch_* < 0.05). C—comparison of CIOA compared to Control, 10 FSH and 10 FSH + CIOA (all: p*_i_* < 0.001; p*_Hoch_* < 0.001). D—10 FSH + CIOA compared to the other groups (all: p*_i_* < 0.05; p*_Hoch_* < 0.05). E—10 FSH compared to CIOA^10 FSH^ (p*_i_* < 0.01; p*_Hoch_* < 0.01). (**4**). Spindle pole width. Categories for M I—tight: <1.70 µm; normal: 3.60 ± 1.90 µm; broad: >5.50 µm. Categories for M II—tight: <1.59 µm; normal: 3.56 ± 1.97 µm; broad: >5.53 µm. (**Top**): Data for M I. A—comparison of Control versus each of the other groups (for all comparisons: p*_i_* < 0.01; p*_Hoch_* < 0.01). B—comparison of CIOA versus 10 FSH, CIOA^10 FSH^ and 10 FSH + CIOA (for all: p*_i_* < 0.01; p*_Hoch_* < 0.05). * CIOA—0.46% oocytes with tight poles in M I. (**Bottom**): Data for M II. C—comparison of Control versus CIOA, 10 FSH, CIOA^10 FSH^ (for all: p*_i_* < 0.01; p*_Hoch_* < 0.01). D—comparison of CIOA versus 10 FSH and 10 FSH + CIOA (for all comparisons: p*_i_* < 0.01; p*_Hoch_* < 0.01). E—comparison of CIOA^10 FSH^ versus 10 FSH + CIOA (p*_i_* < 0.01; p*_Hoch_* < 0.05). (**5**). Presence of tubulin asters. (**Top**): asters in M I. A—each group differs significantly from each other (p_i_ < 0.001; p_*Hoch*_ < 0.01). * Control—1.28% oocytes with cytoplasmic asters in M I. * CIOA^10 FSH^—4.11% oocytes with asters at the spindle poles in M I. (**Bottom**): asters in M II. B—each group differs significantly from the others (p*_i_* < 0.01; p*_Hoch_* < 0.05). ** Control—1.94% oocytes with cytoplasmic asters in M II. ** CIOA^10 FSH^—6.52% oocytes with cytoplasmic asters in M II. (**6**). Presence of a cloud of depolymerized tubulin in the spindle region. (**Left**): presence of a tubulin cloud in M I. A—comparison of CIOA^10 FSH^ versus 10 FSH and 10 FSH + CIOA (for both comparisons: p*_i_* < 0.05; p*_Hoch_* < 0.05). (**Right**): presence of a tubulin cloud in M II. B—comparison of 10 FSH + CIOA versus 10 FSH and CIOA^10 FSH^ (for both comparisons: p*_i_* < 0.001; p*_Hoch_* < 0.001). In all graphs, p*_i_*—Fisher–Freeman–Halton exact test; p*_Hoch_*—correction of p*_i_* for multiple comparisons according to the Hochberg method.

The results concerning spindle size showed a tendency for large spindles and poles in the CIOA group compared to the others, especially to the Control. These differences were significant (Figure 5(1,2,4)). The prevalence of large spindles among CIOA oocytes was striking, approximating or exceeding 2/3 for both M I and M II for all sizes of the spindle (length, width, and pole width).

Concerning spindle length, CIOA oocytes had 2/3 to 3/4 long spindles (Figure 5(1)). For the other FSH-treated groups (10 FSH, CIOA^10 FSH^, and 10 FSH + CIOA), the tendency for spindle elongation relative to the Control was not as pronounced but still significant. Short spindles were only observed in the Control oocytes, while spindles with normal length were an overwhelming majority in all groups except CIOA.

The spindle width and pole size of FSH-treated groups (Figure 5(2,4)) were largely normal, in accordance with the data for the spindle length. The CIOA group oocytes showed considerably larger spindles (wider in nearly 75% and 55% of M I and M II, respectively) with broader poles (more than 72% M I and 63% M II). These data made the CIOA group significantly different from the others. Significant differences in the spindle width were registered between the Control and the CIOA, CIOA^10 FSH^, and 10 FSH + CIOA groups in M I oocytes, whereas in M II, significantly different spindle width was observed in the groups CIOA and CIOA^10 FSH^. The group 10 FSH had a spindle width comparable to that of the Control group, with predominance of the normal category (over 80% for both M I and M II). It nevertheless differed significantly from both Control and the CIOA-treated groups (CIOA, CIOA^10 FSH^, and 10 FSH + CIOA). The 10 FSH + CIOA group had the highest percentage of normal spindle width (over 90% of M I and nearly 87% of M II). This, together with the presence of some slender spindles in addition to some wider ones, distinguishes the group from the other treated groups and makes it similar to Control in both M I and M II. The spindle width roughly corresponded to the spindle length for all of the groups.

The data for spindle pole width (Figure 5(4)) also corresponded to the data for spindle length. The significant pole size differences between the groups (Control, CIOA, 10 FSH, CIOA^10 FSH^ and 10 FSH + CIOA) followed the same main pattern. The Control group had tighter poles than all other groups for both M I and M II. CIOA oocytes were different (with wider spindles and poles) from the others for both M I and M II, while M II oocytes of the 10 FSH + CIOA group were different from CIOA, 10 FSH, and CIOA^10 FSH^ but similar to the Control group.

The tendency of CIOA oocytes toward spindle enlargement was valid for all size parameters: spindle size (length and width) and poles. The FSH-treated groups had considerably higher percentages of long than wide spindles, creating the impression of slender spindles. However, their pole parameters were closer to the data for the spindle length, i.e., the slender spindles usually did not have tight poles. Overall, the Control, 10 FSH, CIOA^10 FSH^ and 10 FSH + CIOA groups had a relatively high proportion of cells with normal spindle size and poles, whereas CIOA differed from them for these characteristics. Strikingly, the group consecutively subjected to both FSH and arthritis (10 FSH + CIOA) had a distribution of spindle size parameters similar to that of the Control group.

The data for spindle integrity are shown in Figure 5(3). It was highest in the Control group (over 86% for M I and almost 62% for M II). In the treated groups, it was reduced to varying degrees: the groups subjected to just one of the studied factors; arthritis or FSH (CIOA and 10 FSH, respectively) had moderate spindle integrity in the range of 40–50%, while the groups influenced by both factors (CIOA^10 FSH^ and 10 FSH + CIOA) had values less than 30% for this parameter. Spindles with detached fibers were observed almost exclusively in groups CIOA and CIOA^10 FSH^, while 10 FSH + CIOA had the highest percentage (over 73%) of totally disorganized spindles.

Spindle integrity was related to two other characteristics: presence of tubulin asters and depolymerization of tubulin in the vicinity of chromosomes. Tubulin asters (Figure 5(5)) were found at the poles in a small minority of Control oocytes. By contrast, two of the treated groups were distinguished by the presence of cytoplasmic and/or polar tubulin asters: CIOA (asters in about 60% of oocytes) and CIOA^10 FSH^ (asters in about 30% of oocytes).

In a proportion of the oocytes, abnormal reaction for tubulin was observed in the equatorial region of the meiotic spindle: microtubules were locally depolymerized and detached from the chromosomes, and an amorphous tubulin cloud surrounded the chromatin. The appearance of this phenomenon is shown in Figure 2E,G, and data about its prevalence are summarized in Figure 5(6). It can be seen that a tubulin cloud is found in all FSH-treated groups, and only in them. The pattern is the same for M I and M II oocytes, with the lowest prevalence in CIOA^10 FSH^ (almost 20%), higher in 10 FSH (about 1/3), and the highest in 10 FSH + CIOA (more than half).

**Figure 6:**
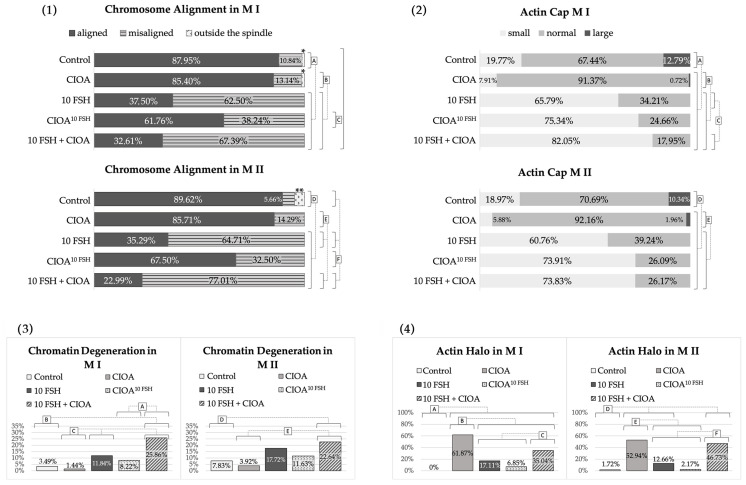
Chromatin and actin parameters in groups influenced by osteoarthritis and/or FSH. (**1**). Chromosome alignment. (**Top**): M I. A—comparison of Control versus 10 FSH, CIOA^10 FSH^ and 10 FSH + CIOA (for all: p*_i_* < 0.001; p*_Hoch_* < 0.001). B—comparison of CIOA versus 10 FSH, CIOA^10 FSH^ and 10 FSH + CIOA (for all: p*_i_* < 0.01; p*_Hoch_* < 0.01). C—comparison of CIOA^10 FSH^ versus Control, CIOA, 10 FSH and 10 FSH + CIOA (for all: p*_i_* < 0.01; p*_Hoch_* < 0.05). * Control—1.20% oocytes with chromosomes that were outside the spindle in M I. * CIOA—1.46% oocytes with chromosomes that were outside the spindle in M I. (**Bottom**): M II. D—comparison of Control versus 10 FSH, CIOA^10 FSH^ and 10 FSH + CIOA (for all: p*_i_* < 0.001; p*_Hoch_* < 0.001). E—comparison of CIOA versus 10 FSH and 10 FSH + CIOA (for all: p*_i_* < 0.01; p*_Hoch_* < 0.01). F—comparison of CIOA^10 FSH^ versus Control, CIOA, 10 FSH, CIOA^10 FSH^ and 10 FSH + CIOA (for all: p*_i_* < 0.01; p*_Hoch_* < 0.01). ** Control—4.72% oocytes with chromosomes that were outside the spindle in M II. (**2**). Comparison of the actin cap size. Categories for M I—small: <49.86 µm; normal: 63.40 ± 13.54 µm; large: >76.94 µm. Categories for M II—small: <49.68 µm; normal: 62.40 ± 12.72 µm; large: >75.12 µm. (**Top**): Data for M I. A—comparison of Control versus each of the other groups (for all comparisons: p*_i_* < 0.01; p*_Hoch_* < 0.01). B—comparison of CIOA versus 10 FSH, CIOA^10 FSH^ and 10 FSH + CIOA (for all: p*_i_* < 0.01; p*_Hoch_* < 0.01). C—comparison of 10 FSH versus 10 FSH + CIOA (p*_i_* < 0.05; p*_Hoch_* < 0.05). (**Bottom**): Data for M II. D—comparison of Control versus each of the other groups (for all comparisons: p*_i_* < 0.01; p*_Hoch_* < 0.05). E—comparison of CIOA versus 10 FSH, CIOA^10 FSH^ and 10 FSH + CIOA (for all: p*_i_* < 0.01; p*_Hoch_* < 0.01). (**3**). Presence of degenerated chromatin. (**Left**): M I. A—comparison between CIOA^10 FSH^ and 10 FSH + CIOA (p*_i_* < 0.01; p*_Hoch_* < 0.05). B—comparison between Control and 10 FSH + CIOA (p*_i_* < 0.01; p*_Hoch_* < 0.01). C—comparison of CIOA versus 10 FSH and 10 FSH + CIOA (for all: p*_i_* < 0.01; p*_Hoch_* < 0.05). (**Right**): M II. D—comparison between Control and 10 FSH + CIOA (p*_i_* < 0.01; p*_Hoch_* < 0.05). E—comparison between CIOA and 10 FSH + CIOA (for all: p*_i_* < 0.01; p*_Hoch_* < 0.05). (**4**). Presence of actin halo. (**Left**): M I. A—comparison of Control versus CIOA, 10 FSH, CIOA^10 FSH^ and 10 FSH + CIOA (for all: p*_i_* < 0.05; p*_Hoch_* < 0.05). B—comparison of CIOA versus 10 FSH, CIOA^10 FSH^ and 10 FSH + CIOA (p*_i_* < 0.01; p*_Hoch_* < 0.01). C—comparison of 10 FSH + CIOA versus 10 FSH and CIOA^10 FSH^ (for all: p*_i_* < 0.01; p*_Hoch_* < 0.05). (**Right**): M II. D—comparison of Control versus CIOA, 10 FSH and 10 FSH + CIOA (for all: p*_i_* < 0.05; p*_Hoch_* < 0.05). E—comparison of CIOA versus 10 FSH and CIOA^10 FSH^ (for all: p*_i_* < 0.001; p*_Hoch_* < 0.001). F—comparison between 10 FSH + CIOA versus 10 FSH and CIOA^10 FSH^ (for all: p*_i_* < 0.001; p*_Hoch_* < 0.001). In all graphs, p*_i_*—Fisher–Freeman–Halton exact test; p*_Hoch_*—correction of p*_i_* for multiple comparisons according to the Hochberg method.

The data about chromosome alignment (Figure 6(1)) showed a high prevalence of well-aligned metaphase plates in both the Control and CIOA groups, which did not differ significantly. The other three groups, i.e., the FSH-treated oocytes, displayed lower percentages of well-aligned metaphases. The difference between them, on the one hand, and the Control and CIOA groups, on the other, was significant. Comparison of Figure 5(6) and Figure 6(1) shows that the higher the prevalence of a tubulin cloud in a group, the higher the proportion of metaphase misalignment. This was not surprising, since the proper attachment of chromosomes to spindle fibers is of critical importance for the quality of metaphase plates.

Figure 6(3) shows the data about chromatin degeneration. As with the study of tubulin cloud and chromosome misalignment, this parameter was elevated in the three FSH-treated groups. Among them, the percentage of degenerated chromatin was lowest in the CIOA^10 FSH^, moderate in 10 FSH and highest in 10 FSH + CIOA. Statistically significant differences were found between Control and 10 FSH + CIOA, and between CIOA^10 FSH^ and 10 FSH + CIOA for both M I and M II oocytes. CIOA oocytes compared to 10 FSH and 10 FSH + CIOA differed significantly for the M I oocytes only.

The microfilament cytoskeleton was evaluated by the actin cap size and the presence of an actin halo. Normal cap size predominated in the Control group (Figure 6(2)). The CIOA group also showed an overwhelming majority of normal-sized caps. In FSH-treated groups, the most common pattern was the presence of small actin caps for both M I and M II, observed in 60–82% of oocytes depending on the group. Large caps were not observed in these groups at all. Statistically significant differences were observed between the Control and all of the other groups, as well as between CIOA and the FSH-treated groups.

The actin halo was very rare in the Control group, observed in less than 2% of cells in M II and none of those in M I (Figure 6(4)). By contrast, halo was observed in the majority of CIOA oocytes and a substantial proportion (over 1/3 for M I and nearly half for M II) of 10 FSH + CIOA. The other two groups (10 FSH and CIOA^10 FSH^) showed intermediate results. The differences between the Control and all other groups were significant for both M I and M II. For M I, each of CIOA and 10 FSH + CIOA also differed significantly from all other groups, and for M II, they differed significantly from 10 FSH and CIOA^10 FSH^ but not from each other.

Overall, the groups for the study of the influence of osteoarthritis and/or FSH (Control, CIOA, 10 FSH, CIOA^10 FSH^ and 10 FSH + CIOA) were characterized by similarity with CIOA^Nut^ of peculiarities related to CIOA but also by the unique influence of FSH alone or in combination with osteoarthritis. Tubulin asters were found not only in the cytoplasm (as in estradiol-treated groups) but also at spindle poles. The FSH-treated groups had a tendency toward a depolymerized tubulin cloud, chromosome misalignment and/or degeneration, as well as small actin caps, though there was some inter-group variation in these parameters. When both factors simultaneously influenced oogenesis (the group CIOA^10 FSH^), chromosome misalignment and degeneration were lower than in other FSH-treated groups. The maturation rate was decreased most in the CIOA group. The FSH-treated groups had a slightly better maturation rate but were characterized by a common presence of transitional and post-M II stages.

## 4. Discussion

### 4.1. Oogenesis with Osteoarthritis in the Background

Oogenesis in the presence of osteoarthritis is a problem that has not yet been adequately studied. To our knowledge, our team has been the first to specifically address it. Our study has certain limitations, including the following: lack of direct knowledge about the reproductive potential of the mice and their oocytes because neither in vitro fertilization nor natural conception was performed; a single arthritis model was used; and the observations of cell structures were performed at the light microscopic level.

Using the CIOA mouse model, which provides an opportunity to analyze oocytes matured during the established phase of osteoarthritis, we investigated first the impact of the disease process on the oocyte maturation rate, cytoskeleton, and chromatin, and then the effects of different reproductive hormone regimens. We have described the influence of osteoarthritis *per se* elsewhere [48], and here we are using the respective group (CIOA) only to compare its peculiarities to those of the FSH-treated groups. The results for the estradiol-treated groups were compared to those for a corresponding group (CIOA^Nut^) kept on the same diet.

Oocytes from the groups with “unmedicated” osteoarthritis (CIOA and CIOA^Nut^) showed a substantial increase in all sizes of the meiotic spindle: length, width, and pole width. In addition, tubulin asters were observed in most cells. The presence of tubulin asters is a prerequisite for meiotic spindle formation during germinal vesicle breakdown (GVBD), but during the subsequent stages of meiosis, these nascent microtubules decrease in both number and length. Hence, the increased size and abnormal nucleation of microtubules indicated that spindle morphogenesis in the CIOA mice was affected by the disease process.

According to literature data, enlargement of the oocyte meiotic spindle and its poles has been observed after injection of antibodies against the small GTPase Ran [51], upregulation of the motor protein Eg5 (kinesin-5) [52], and treatment with the microtubule-stabilizing drug taxol, which also induces microtubule nucleation [51,53]. The coincidence of the two findings in our oocytes, spindle enlargement and presence of asters, is in accordance with literature data about the influence of microtubule nucleation on spindle size. There are reports that in proliferating somatic cells, mitotic spindle size increases in parallel with the increase in the number and/or activity of microtubule nucleation centers [54]. In maturing oocytes, this dependency is even stronger, indicating that microtubule nucleation becomes the dominant factor determining meiotic spindle size. It is supposed that this is due to the large cytoplasmic volume of oocytes [55]. Thus, we can hypothesize that both the large spindle size and the presence of asters resulted from some osteoarthritis-related change(s) that abnormally activated microtubule nucleation in maturing oocytes.

The actin cytoskeleton was also affected by experimental osteoarthritis. While the actin cap had a generally normal appearance, more than half of the oocytes displayed, in addition to it, a diffuse actin halo in the ooplasm. Since this halo is neither a normal structure nor a common defect, its potential impact on oocyte maturation and functions is not known at present.

It should be noted that the majority of oocytes in the CIOA and CIOA^Nut^ groups had normal chromosome alignment, indicating that their large spindles had successfully arranged the chromosomes in a metaphase plate. However, this metaphase was in most cases metaphase I, implying activation of the spindle checkpoint and meiotic arrest. The defective oogenesis in the CIOA and CIOA^Nut^ groups compared to the Control and Control^Nut^, respectively, could be explained by systemic inflammatory factors, making the ovarian microenvironment less capable of supporting normal oocyte maturation.

### 4.2. Effects of Estradiol on Cytoskeleton and Chromatin of the Oocytes

The pronounced increase in osteoarthritis incidence in postmenopausal women, combined with data from ovariectomized animal models, suggests that ovarian deficiency predisposes to the disease [32,33]. Recent studies have found an anti-arthritic effect of estradiol both on women with osteoarthritis [34] and in the CIOA mouse model [45,46,47]. While these data are not yet the scientific consensus, and estradiol is currently not used for treatment of osteoarthritis except in experimental settings, it is appropriate to study the influence of exogenous estradiol on oogenesis in osteoarthritis, given that a small but significant minority of patients with osteoarthritis are reproductive-age women, more than 4% in some recent studies [56]. In physiological concentrations, estrogen is essential for oogenesis and folliculogenesis [57]. However, this does not mean that an eventual estrogen supplementation for treatment of osteoarthritis would be beneficial or even neutral for reproductive functions, because dose and timing are of paramount importance for the biological activities of hormones. To collect relevant data, we studied the combined effect of osteoarthritis and estradiol on oogenesis in the CIOA mouse model.

It should be noted that oocytes of Control^Nut^, the control of the estradiol-treated groups receiving hazelnut-cocoa cream, showed a slightly better quality of tubulin and actin cytoskeleton than oocytes from the pure Controls that received only standard food for rodents. Similarly, the CIOA^Nut^ had better spindle integrity than CIOA. We suppose that some nutritional ingredient contained in the hazelnut-cocoa cream had positively affected the oogenesis of Control^Nut^ relative to Control, and of CIOA^Nut^ relative to CIOA. The available data give no specific clue about the nature of this component, but it is known that the Nutella spread is rich in fats and carbohydrates, which could affect the metabolism of steroid hormones. More generally, the difference between the two control groups underscored the importance of carefully planning adequate controls in experimental studies, since unexpected factors (such as the use of Nutella) could affect the outcome.

The results from the experimental estradiol-treated groups, however, showed that the benefit of adding Nutella could not outweigh the harmful effects caused by other factors. Estradiol, a key hormone in the follicular phase of the female reproductive cycle when oocyte meiotic maturation takes place, disrupted the maturation of our oocytes, resulting in a reduced maturation rate and the presence of degenerated oocytes. The majority of oocytes had cytoplasmic tubulin asters and undersized actin caps. While the mechanism underlying the reduction in the actin cap size in our study is unknown, there are reports of specific molecular factors that can induce such a change, e.g., inhibition of myotonic dystrophy kinase-related Cdc42-binding kinase (MRCK) [58]. We can hypothesize that the small actin caps of our oocytes could result from similar disturbances of the cell cycle control system, leading to deficient activation of ring myosin II and thus reduction in the area which is occupied by the actin nucleator Arp2/3 and determines the actin cap size. The suboptimal outcome of the oogenesis in the E2 group is in accordance with human studies where the best outcomes of in vitro fertilization have been obtained with serum estradiol per oocyte ratio within a certain interval, and an increase in this ratio above the optimum values was associated with a decline in pregnancy rates [59].

When osteoarthritis and estradiol treatment were concurrent, oocyte quality was most affected. Mice with CIOA treated with estradiol (CIOA + E2^Nut^ group) had the highest proportion of pre-M I oocytes, the broadest M II spindles and poles, and the highest proportion of oocytes with cytoplasmic tubulin asters. As discussed above about the influence of CIOA, which was also associated with persistent tubulin asters and large spindles, the presence of asters may also be related to the formation of elongated spindles with broad poles typical of these oocytes. In other words, the increased number and/or activity of microtubule-organizing centers, manifested as tubulin asters, could underlie the observed meiotic spindle enlargement. In the CIOA + E2^Nut^ group, these peculiarities were most pronounced, indicating that the effects of osteoarthritis and estradiol were additive in this respect.

The disturbance of microtubule organization could also explain the poor maturation rate characteristic of the CIOA + E2^Nut^ group. Recent data indicate that cytoplasmic asters slow down the movement of oocyte meiotic spindle towards the periphery during metaphase I, and their decrease by autophagy allows spindle migration to the cortex; autophagy inhibitors lead to their persistence, delayed spindle migration, and enlarged polar bodies [60]. In this respect, oocytes from our CIOA + E2^Nut^ group resembled the cells with suppressed autophagy. While the large polar bodies described by these authors were not observed in the present study, there was a reduced maturation success and an increased proportion not only of metaphase I but also of the preceding highly immature stages, as well as of degenerated oocytes. This suggests impaired meiotic maturation for this group, presumably due to activation of the spindle checkpoint, and perhaps also of earlier checkpoints.

The alignment of chromosomes in the meiotic spindles under the combined influence of CIOA and estradiol, however, remained relatively normal, and for M I exceptionally good. We can assume that in other groups, the spindle assembly checkpoint is activated more often by disruption in the connection of chromosomes to the spindle, and in the CIOA + E2^Nut^ group by the disrupted construction of the spindle itself.

The actin cap was also affected by estradiol treatment. Without induction of osteoarthritis (the E2^Nut^ group), it showed a reduced size, indicating some disruption in the organization of cortical microfilaments. Curiously, this effect was far less pronounced in the CIOA + E2^Nut^ group subjected to both CIOA and estradiol, though the chromatin, which is important for actin cap formation, had a relatively high rate of degeneration in this group. It is not clear whether an undersized actin cap could still perform its functions related to anchoring of the meiotic spindle and cytokinesis.

Overall, most parameters of the estradiol-treated groups were worse than in their counterparts without exogenous estradiol. This is not surprising, since each hormone is expected to work best at its physiological concentration, and at elevated levels to exhibit undesirable effects. If we extrapolate the results from the particular mouse model, we can assume that if the beneficial effects of estradiol on osteoarthritis found by some teams are confirmed, and estradiol supplementation becomes standard treatment for women with the condition in the future, this is not expected to improve their oogenesis; moreover, its further deterioration is possible.

### 4.3. Effects of FSH on Cytoskeleton and Chromatin of the Oocytes

Follicle-stimulating hormone is part of the natural gonadotropic regulation of ovarian function by the pituitary gland. This pathway is used in assisted reproduction: in order to achieve ovarian stimulation and production of an increased (compared to the normal for the species) number of dominant follicles, exogenous FSH is administered. It supports multiple simultaneously growing follicles and ensures a higher yield of preovulatory follicles. However, this approach carries a risk of ovarian hyperstimulation and impaired oocyte quality with compromised chromosome segregation and an increased rate of aneuploidies. Such results have been described in humans as well as various animal models, and have led to accepting milder stimulation protocols [61,62,63].

When a woman with a preexisting condition is undergoing ovarian stimulation as part of assisted reproduction, two questions arise: about the influence of the stimulation on the condition, and about the influence of the condition on the outcome of the stimulation. We have previously addressed the first question, using the same model, and have found a negative impact of FSH on important CIOA characteristics: it increased cartilage degradation and the number of osteoclasts [45]. In the present study, we sought an answer to the second question about the effects of FSH on oogenesis with underlying osteoarthritis.

As expected, the groups treated with FSH were characterized by a significantly higher yield of oocytes for analysis. However, the quality of the cells was different from both the control groups and the groups analyzed only for the effect of CIOA. The maturation rate was reduced, i.e., proportionally fewer M II and more M I oocytes were observed than in the other groups. Moreover, there was a relatively high percentage of degenerated cells, and a significant percentage of cells were found in transition from the first to the second meiotic division, as well as in an unusual state for ovulated oocytes, in anaphase or telophase of the second meiotic division, which should be part of the fertilization process.

With regard to the latter finding, there are reports in the literature of unfertilized mammalian oocytes that proceed beyond metaphase II. This event, formerly considered a parthenogenetic activation, is observed in defective, damaged, or aged oocytes and has been described in recent sources as apoptosis [64,65,66]. The cause of the transition of dying metaphase cells to anaphase and telophase is that the completion of apoptosis, for unclear reasons, requires an exit from M phase. Mitotic cells in which apoptosis is triggered also proceed to the final stages before dying, a phenomenon called mitotic catastrophe [67]. Based on the literature data and our results, it can be concluded that exogenous FSH, applied according to the described protocol, induces apoptosis in a proportion of oocytes.

The characteristics of the meiotic spindles and the chromosome alignment showed specific defects different from all other analyzed groups. A characteristic feature of the oocytes from the FSH groups was the common presence of a cloud of depolymerized tubulin in the equatorial region. This anomaly was most frequent in the 10 FSH and 10 FSH + CIOA groups, and correlated with the high percentage of misaligned chromosomes in these oocytes. The FSH-treated groups had the highest percentage of disorganized spindles, which in 10 FSH + CIOA reached about 3/4 of the analyzed cells. The proportion of cells with degenerated chromatin was also highest in this group. The CIOA^10 FSH^ group, despite the spindle defects, had a relatively good arrangement of metaphase chromosomes compared to the other two FSH-treated groups. This implies that the temporal course of the FSH treatment relative to the CIOA progression was important for the nature and degree of the changes in oogenesis.

The abnormalities of the meiotic spindles discussed above were accompanied by the smallest actin caps, significantly smaller than in the Control. Given the importance of chromatin for the actin cap organization through a Ran-mediated pathway, we can hypothesize that the small cap size reflected the poor condition of chromatin. Actin halo was rarely found in groups 10 FSH and CIOA^10 FSH^, but was present in a substantial proportion of the oocytes of group 10 FSH + CIOA, again reflecting the importance of whether FSH preceded or was simultaneously applied with CIOA.

It is logical to assume that problems in spindle organization would disrupt the alignment of the chromosomes and their future segregation. However, in the CIOA^10 FSH^ group, good chromosome alignment was observed, despite a high percentage of disorganized spindles and spindles with detached fibers (unlike the other two FSH groups). These results imply that at least some of the spindle defects, and probably some of the general cytoskeletal abnormalities, may be related to damage occurring after the assembly of the meiotic spindle and arrangement of the chromosomes in a metaphase plate. However, when present at the time of ovulation, such defects would not allow successful completion of meiosis and would thus make the oocyte incapable of fertilization.

The results from the FSH groups show that obtaining a higher number of oocytes is at the expense of deteriorated quality of meiotic spindles and metaphase plates. The main function of FSH in female reproduction is to stimulate and support the development of preantral and antral follicles, and its exogenous administration alone, before or parallel to CIOA, resulted in the development of a greater than the species-specific number of follicles per reproductive cycle. The exogenously added FSH in the present study, although limited to a 10-day period, represented a uniform and relatively long-term treatment with respect to the reproductive period in mice. Combined with the stimulation for oocyte retrieval, the FSH action was cumulative for the duration of the experiment.

Other authors have reported that excessive FSH stimulation in a heifer model resulted in abnormalities in the differentiation of granulosa and cumulus cells, differences in the size of preovulatory follicles, and premature luteinization [62]. Studies of the effects of increased FSH levels during in vitro maturation of human oocytes have found abnormalities in meiotic chromosome segregation [61]. Notably, the results of the present study concerning the groups 10 FSH and 10 FSH + CIOA showed not a significantly elevated proportion of immature oocytes in M I but, rather, a high percentage of oocytes that were in transitional stages between M I and M II, or had exited the metaphase II arrest. The latter cells were most likely undergoing apoptosis, possibly as a result of cytoskeletal abnormalities. About the oocytes in transitional stages (Ana I/Telo I), based on their low but non-zero prevalence in other groups, we can suppose that some were indeed in transition, but most were undergoing apoptosis that started in M I. It is notable that degenerated oocytes were also relatively common in the FSH-treated groups. In particular, FSH treatment prior to osteoarthritis induction led to uniquely severe cytoskeletal abnormalities that presumably negate the fertilization and development potential of affected oocytes. This finding could have implications for women who develop osteoarthritis after undergoing hormonal stimulation for assisted reproduction. Epidemiological studies on osteoarthritis risk after assisted reproduction are so far absent from the published literature, and given the increasing use of in vitro fertilization and related techniques, this is an appropriate topic for future research.

The treatment of animals with FSH in order to assess its effects on oogenesis revealed a negative impact of exogenous FSH. The obtained results showed impaired oocyte quality, defects in their meiotic maturation, and a tendency to degeneration and apoptosis. Considering the possibilities that some female patients with osteoarthritis will seek assisted reproduction, the data obtained using our CIOA model suggest that mild hormonal stimulation protocols may be preferable for them in order to minimize eventual deleterious effects of FSH on both their oogenesis and the underlying condition.

## 5. Conclusions

Osteoarthritis without exogenous hormonal influence led to enlarged meiotic spindles and the presence of tubulin asters in most of the oocytes, indicating abnormal microtubule nucleation and spindle morphogenesis in the CIOA mice as a result of the disease process. The actin cap was generally normal, but there was often a diffuse actin halo in the ooplasm, which is not a common finding. Most oocytes had normal chromosome alignment, indicating that their large spindles had successfully assembled and then arranged the chromosomes. However, the increased rate of M I oocytes implied activation of the spindle checkpoint and meiotic arrest. The defective oogenesis in the groups with CIOA could be explained by disease-induced systemic inflammatory factors brought to the ovary by circulation, making the ovarian microenvironment less capable of supporting normal oocyte maturation.

When CIOA was compounded by estradiol, most parameters were worse than in their counterparts without exogenous estradiol, though the metaphase plates were generally well aligned. Estradiol, similarly to CIOA, led to a decrease in maturation rate, spindle enlargement, and tubulin asters but had the opposite influence on the actin cap, reducing its size. Combining both factors led to additive effects, an actin cap with average appearance, but the worst maturation rate and the highest percentage of degenerated cells. Hence, eventual estradiol treatment of osteoarthritis is not expected to be beneficial for oogenesis.

In FSH-treated CIOA groups, tubulin asters were found not only in the cytoplasm (as in estradiol-treated groups) but also at spindle poles. This, however, was not accompanied by spindle enlargement. There was a tendency of FSH-treated oocytes toward a depolymerized tubulin cloud, chromosome misalignment and/or degeneration, and diminished actin cap, as well as a common presence of transitional and post-M II stages indicative of apoptosis; these effects were aggravated when FSH treatment was followed by CIOA induction. These cytoskeletal and chromatin abnormalities presumably disturb the fertilization and development potential of affected oocytes, implying a need for precise adjustment of the doses of FSH used in assisted reproduction in cases of patients with osteoarthritis.

## Figures and Tables

**Table 1:** Numbers and percentages by stage of oocytes influenced by osteoarthritis and/or estradiol combined with Nutella, and their control group *.

Groups	Total Number	Before M I Number (%)	M I Number (%)	Ana I/ Telo I Number (%)	M II Number (%)	Ana II/Telo II Number (%)	D Number (%)
Control^Nut^	93	2 (2.15%)	40 (43.01%)	1 (1.08%)	49 (52.69%)	0	1 (1.08%)
CIOA^Nut^	111	0	59 (53.15%)	2 (1.80%)	48 (43.24%)	0	2 (1.80%)
E2^Nut^	81	2 (2.47%)	36 (44.44%)	4 (4.94%)	32 (39.51%)	0	7 (8.64%)
CIOA + E2^Nut^	55	17 (30.91%)	12 (21.82%)	0	12 (21.82%)	0	14 (25.45%)

* Categories used: “Before M I”—GV/GVBD stages (earlier than Metaphase I); “M I”—Metaphase I; “Ana I/Telo I”—Anaphase I or Telophase I stages; “M II”—Metaphase II; “Ana II/Telo II”—stages after Metaphase II (Anaphase II or Telophase II); “D”—degenerated oocytes. The numbers outside brackets are absolute, and those in brackets are percentage proportions of the total.

**Table 2:** Numbers and percentages by stage of oocytes influenced by osteoarthritis and/or FSH, and their control group *.

Groups	Total Number	Before M I Number (%)	M I Number (%)	Ana I/ Telo I Number (%)	M II Number (%)	Ana II/Telo II Number (%)	D Number (%)
Control	198	1 (0.51%)	83 (41.92%)	1 (0.51%)	111 (56.06%)	0	2 (1.01%)
CIOA	193	2 (1.04%)	139 (72.02%)	0	51 (26.42%)	0	1 (0.52%)
10 FSH	214	4 (1.87%)	76 (35.51%)	23 (10.75%)	79 (36.92%)	16 (7.48%)	16 (7.48%)
CIOA^10 FSH^	139	2 (1.44%)	73 (52.52%)	3 (2.16%)	46 (33.09%)	4 (2.88%)	11 (7.91%)
10 FSH + CIOA	286	3 (1.05%)	117 (40.91%)	27 (9.44%)	107 (37.41%)	24 (8.39%)	8 (2.80%)

* Categories used: “Before M I”—GV/GVBD stages (earlier than Metaphase I); “M I”—Metaphase I; “Ana I/Telo I”—Anaphase I or Telophase I stages; “M II”—Metaphase II; “Ana II/Telo II”—stages after Metaphase II (Anaphase II or Telophase II); “D”—degenerated oocytes. The numbers outside brackets are absolute, and those in brackets are percentage proportions of the total.

## Data Availability

The original contributions presented in this study are included in the article. Further inquiries can be directed to the corresponding author.

## Supplementary Materials

The following supporting information can be downloaded at: https://www.mdpi.com/article/10.3390/biomedicines14040857/s1, Table S1: Names of oocyte groups according to the regimen of treatment of animals from which they were obtained.

## Abbreviations

The following abbreviations are used in this manuscript:

BSA	Bovine serum albumin
CIOA	Collagenase-induced osteoarthritis
E2	Estradiol
FITC	Fluorescein isothiocyanate
FSH	Follicle-stimulating hormone
GTPase	Guanosine triphosphatase
GV	Germinal vesicle
GVBD	Germinal vesicle breakdown
ICR	Institute of Cancer Research
IgG	Immunoglobulin G
IU	International unit
LSCM	Laser-scanning confocal microscopy
MDPI	Multidisciplinary Digital Publishing Institute
M I	Metaphase first
M II	Metaphase second
PBS	Phosphate-buffered saline
TRITC	Tetramethylrhodamine isothiocyanate
