# Supplementary material for: Modulation Effects of Reproductive Hormones on Oogenesis in a Collagenase-Induced Osteoarthritis Mouse Model
Source: Biomedicines. 2026 Apr 9;14(4):857. doi: 10.3390/biomedicines14040857 (PMC13114014; doi:10.3390/biomedicines14040857)
Supplement: Supplementary file 1 [file biomedicines-14-00857-s001.zip › biomedicines-4168335-supplementary.pdf]

Table S1. Names of oocyte groups according to the regimen of treatment of animals from which they were obtained.

Group	Treatment
Control	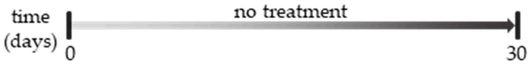
Control^{Nut}	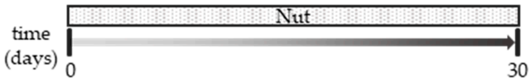
CIOA	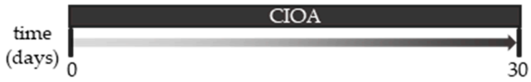
CIOA^{Nut}	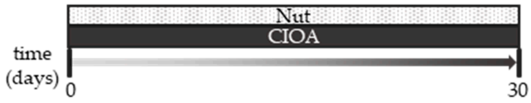
E2^{Nut}	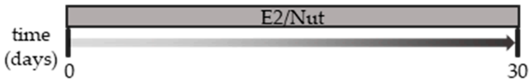
CIOA+E2^{Nut}	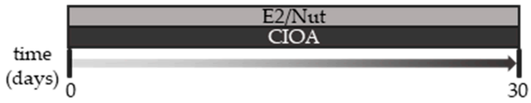
10 FSH	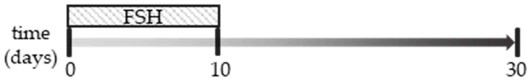
CIOA$^{10\ FSH}$	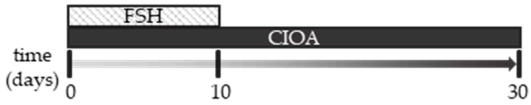
10 FSH+CIOA	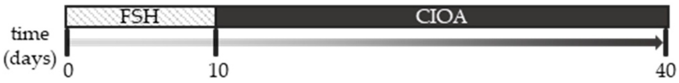
